# Relationship between clinical outcomes measures and personal and social performance functioning in a prospective, interventional study in schizophrenia

**DOI:** 10.1002/mpr.1855

**Published:** 2020-12-23

**Authors:** Roland Vauth, Bernardo Carpiniello, Jacek Turczyński, Mikhail Ivanov, Pierre Cherubin, Marjolein Lahaye, Andreas Schreiner

**Affiliations:** ^1^ Center for Mental Health Department of Psychiatry and Psychotherapy University Hospital of Psychiatry Basel University of Basel Basel Switzerland; ^2^ Unit of Psychiatry Department of Medical Sciences and Public Health University of Cagliari Cagliari Italy; ^3^ Department of Developmental Psychotic, and Geriatric Psychiatry Medical University of Gdańsk Gdańsk Poland; ^4^ St Petersburg Psychoneurological Research Institute St Petersburg Russia; ^5^ Medical Affairs EMEA Janssen‐Cilag Issy‐les‐Moulineaux France; ^6^ Medical Affairs EMEA Janssen‐Cilag BV Breda Netherlands; ^7^ Medical & Scientific Affairs EMEA Janssen‐Cilag GmbH Neuss Germany

**Keywords:** clinical outcomes, psychosocial functioning, schizophrenia

## Abstract

**Objectives:**

To explore clinical and demographic characteristics impacting patient functioning by determining extent of overlap in factors driving change in Personal and Social Performance (PSP) and other clinical outcomes.

**Methods:**

Post‐hoc analysis from a single‐arm trial of paliperidone extended release in adult patients with nonacute symptomatic schizophrenia. Psychosocial functioning measures: PSP, Clinical Global Impression–Severity (CGI‐S), Positive and Negative Syndrome Scale (PANSS), Short‐Form 36 (SF‐36), treatment satisfaction, sleep quality/daytime drowsiness, and Extrapyramidal Symptoms Rating Scale.

**Results:**

Highest correlations with PSP total score change included PANSS total score change (Spearman's *r* = 0.607), PANSS general psychopathology change (*r* = 0.579), and CGI‐S change (*r* = 0.569). A PSP score change of −32 predicted 90% probability of deterioration in CGI‐S (score change of ≥1). The power of PSP change to predict PANSS total score change was lower. Linear stepwise regression demonstrated independent relationships for PSP change and: PANSS total change; CGI‐S change; SF‐36 Mental Component change; treatment satisfaction at endpoint; PSP at baseline; previous psychiatric hospitalizations. *R* ^2^ = 0.55 meant that 45% of PSP variation could not be explained by other clinical outcome measures.

**Conclusions:**

Psychosocial functioning improvement is important in schizophrenia. PSP may be valuable for assessing functioning; it encompasses psychosocial and clinical factors not measured by other established assessments.

## 1 INTRODUCTION

Schizophrenia is a chronic heterogeneous disease with a variety of positive, negative, and associated symptoms. Functional deficits across multiple symptom domains are a core characteristic of the disease, and are essential for a diagnosis of schizophrenia (American Psychiatric Association Association, 2013). In a recent systematic literature review, schizophrenia was rated as the most disabling of the 11 mental disorders assessed (Eaton et al., 2008). It affects multiple aspects of patient functioning, including occupational and social functioning and independent living skills (Harvey & Bellack, 2009; Harvey, Green, Keefe, & Velligan, 2004). For instance, two‐thirds of patients with schizophrenia cannot perform basic social roles (such as acting as a spouse or parent), even during periods of remission (Bellack et al., 2007; Haro et al., 2011), and less than one‐third are employed; those who are employed tend to have low‐paying or less‐skilled employment compared with their premorbid functioning (Bellack et al., 2007; Haro et al., 2011). Poor functioning, therefore, results in a considerable economic impact and a substantial social burden on society and patients themselves (Bellack et al., 2007; Sun, Liu, Christensen, & Fu, 2007; Wu et al., 2005).

Adequate social functioning is essential for patients with schizophrenia, as it is for any individual, as it helps them to achieve their life goals. It is, of course, important to note that patients with multiple psychotic symptoms may still have good level of functioning, indicating a relative independency of symptoms and social functioning. Other characteristics that were correlated with better functioning in clinical studies among patients with schizophrenia are neurocognition, resilience, and integration of recovery style in daily life (Poloni et al., 2018; Zizolfi et al., 2019) as well as social cognition and self‐stigmatizing (Morin & Franck, 2017).

The majority of clinicians recognize that improved personal and social functioning are important treatment goals, and most assess the personal and social functioning of their patient at each visit (Gorwood et al., 2013). Progress made in the pharmacological and nonpharmacological treatment of schizophrenia (including the introduction of atypical antipsychotics) has resulted in clinical attention being focused on improving psychosocial functioning, cognition, and negative symptoms, in addition to controlling positive symptoms of psychosis and improving side‐effect profiles (Karow & Naber, 2002). Regular systematic measurement of functioning should, therefore, receive more attention when assessing people with schizophrenia in routine clinical care.

Despite the increased focus on the improvement of social and personal functioning as a treatment target, more than 80% of clinicians report that they determine the level of patient functioning through clinical interview rather than using a specific assessment scale (Gorwood et al., 2013). In addition, in a recent literature review of 301 studies that assessed social functioning, 87 separate outcome measures were described, with a significant lack of data on psychometric properties (Burns & Patrick, 2007). These findings highlight the need for standardization of the measurement of social and personal functioning in schizophrenia, and the need for a specialized measurement tool.

Existing scales are considered to be too complex for day‐to‐day practice as they assume too high a level of functioning (evaluated using the Level of Functioning scale) (Brissos, Molodynski, Dias, & Figueira, 2011; Figueira & Brissos, 2011), confound psychosocial functioning with psychopathological symptoms (evaluated using the Global Assessment of Functioning scale); or are not clearly operationalized (evaluated using the Social and Occupational Functioning Assessment Scale, which does not have anchors of rating in its definition) (Morosini, Magliano, Brambilla, Ugolini, & Pioli, 2000). The Personal and Social Performance (PSP) scale was developed specifically to measure the psychosocial functioning of psychiatric patients during the course of treatment (Morosini et al., 2000). This scale was conceptualized by building on previously used measures for psychosocial function (Juckel & Morosini, 2008). Outcome scores range from 1 to 100, and address four main domains of social functioning: (1) socially useful activities, (2) personal and social relationships, (3) self‐care, and (4) disturbing and aggressive behavior. The PSP scale has been validated in both stable and acutely ill patients, and is sensitive to effects of treatment with pharmacologically efficacious agents (Huang et al., 2012; Nasrallah, Morosini, & Gagnon, 2008; Patrick et al., 2009). Relationships have been confirmed between the PSP and other clinical scales (including the Positive and Negative Syndrome Scale [PANSS] and the Clinical Global Impression–Severity [CGI‐S] scale) and hospitalization (Hough et al., 2009; Kozma, Dirani, Canuso, & Mao, 2010; Patrick et al., 2009).

In this post‐hoc analysis of clinical trial data, we analyzed the relationship between patient PSP scores, baseline characteristics, and clinical variables assessed by various well‐established measurement scales. The objective of this study was to explore key clinical and demographic characteristics that impact patient functioning, and to assess the PSP scale's utility as the primary scale for clinicians assessing social function by determining the extent of overlap in factors driving change in PSP and other clinical outcome measures.

## 2 METHOD

### 2.1 Study design and patients

The data for this analysis came from a 6‐month, prospective, multicentre, interventional, single‐arm study, conducted between April 2007 and January 2009 (Schreiner et al., 2014). The study included patients aged ≥18 years with nonacute but symptomatic schizophrenia, who had previously been unsuccessfully treated with oral antipsychotics. The primary study included 1812 patients who had a PSP baseline score; only patients with both baseline and 6‐month endpoint PSP scores (*n* = 1646) were included in this analysis. Baseline demographic and clinical characteristics of this patient population are summarized in Table 1.

**TABLE 1:** Baseline demographics and clinical characteristics

Characteristics	*N* = 1812	
Gender, *n* (%)		
Male	1086 (59.9)	
Female	726 (40.1)	
Age, years		
Mean (SD)	40.1 (12.6)	
Range	17–93	
Types of schizophrenia, *n* (%)^a^		
Paranoid	1373 (75.8)	
Undifferentiated	213 (11.8)	
Residual	123 (6.8)	
Disorganized	88 (4.9)	
Other	14 (0.8)	
Age at schizophrenia milestones, years, mean (SD)		
Onset of symptoms	27.3 (9.5)	
Onset of antipsychotic treatment	28.7 (9.8)	
Diagnosis of schizophrenia	30.0 (10.2)	
Hospitalized at baseline or start of study, *n* (%)		
No	1472 (81.2)	
Yes	340 (18.8)	
PANSS total score, mean (SD)	79.3 (20.4)	
Body weight, kg, mean (SD)	81.0 (17.9)	
Body mass index, kg/m^2^, mean (SD)	27.5 (5.6)	
Commonly used (>5%) previous antipsychotic medications	*n* (%)	Mean (SD) daily dose, mg
Risperidone	694 (38.3)	4.3 (2.3)
Olanzapine	396 (21.9)	14.2 (7.5)
Haloperidol	191 (10.5)	10.1 (8.4)
Quetiapine	173 (9.5)	485.3 (279.0)
Aripiprazole	141 (7.8)	19.4 (11.4)
Amisulpride	135 (7.5)	551.9 (352.1)
Commonly used (>5%) psychotropic drugs, %		
Benzodiazepines	36.9	
First‐generation tricyclic antidepressants	20.9	
Selective serotonin reuptake inhibitors	15.6	
Other antidepressants	8.7	
Zolpidem and zopiclone	8.2	
Nonselective monoamine reuptake inhibitors	5.1	

Abbreviations: PANSS, Positive and Negative Syndrome Scale; SD, standard deviation.

^a^ Based on an evaluable sample of 1811 patients.

### 2.2 Treatment

All patients were treated with open‐label, flexibly dosed paliperidone extended release (ER) (3–12 mg/day); selection of the initial dose and dose adjustments for individual patients were made at the discretion of the clinician. Other antipsychotics for the treatment of schizophrenia were prohibited during the study. Previous antipsychotics prescribed for schizophrenia were discontinued or tapered, with the tapering schedule preferably completed within 4 weeks. Mean (standard deviation) daily doses of previous antipsychotics are shown in Table 1.

### 2.3 Measurement scales

Psychosocial functioning was measured using the PSP scale (Table S1). Other scales used to measure clinical symptoms and disease severity were: the CGI‐S scale (range 1–7; Guy, 1979), which is designed for clinicians to assess positive, negative, depressive and cognitive symptoms in schizophrenia; the PANSS total score (range 7–210; Kay, Fiszbein, & Opler, 1987; Kay, Opler, & Lindenmayer, 1989), a clinician‐reported survey designed to measure schizophrenia symptom severity; the PANSS subscales (positive, negative, general psychopathology, Marder factors; Daniel, 2013; Marder, Davis, & Chouinard, 1997; van Erp et al., 2014); the physical component and mental component of the Short‐Form (SF‐36) Health Survey, a patient‐reported survey of their general health, scored using norm‐based methods using general US population factors (RAND.org); treatment satisfaction (using a 5‐point categorical scale, ranging from 1 [very good] to 5 [very poor]); sleep quality (over the previous 7 days, using an 11‐point categorical scale, ranging from 0 [very badly] to 10 [very well]); daytime drowsiness (over the previous 7 days, measured from 0 [not at all] to 10 [all the time]); and extrapyramidal symptoms using the Extrapyramidal Symptoms Rating Scale (ESRS) total score (range 0–102) (Table S1) (Chouinard & Margolese, 2005).

### 2.4 Statistical analyses

Unless otherwise stated, all analyses were performed on change scores from baseline to the 6‐month endpoint (last observation carried forward). Scatterplots and Spearman's correlation coefficients were used to assess the relationship between change in PSP and change in other scale measures. The predictive power of the PSP scale to capture changes in disease severity (CGI‐S) was assessed using logistic regression analysis with dichotomized CGI‐S (≥1‐point increase vs. no change/decrease) as the dependent variable, and PSP change as the independent variable. A discriminant analysis with PSP change as the independent variable and change in CGI‐S trichotomy (decrease, increase, or no change in score) was also carried out. A similar discriminant analysis was done with a trichotomized PANSS total score as the independent variable (a decrease of >20 points, a decrease of ≤20 points, and no change or increase). For the discriminant analysis, the decision was taken to use absolute PANSS total score changes instead of relative changes because the percent changes were highly skewed and difficult to interpret. A decrease of 20 points was considered relevant in this nonacute population.

Some stepwise linear regression analyses were performed to investigate whether the change in PSP score could be predicted by the change in other known clinical scale measures (PANSS score, CGI‐S, etc.). Sensitivity analyses using similar stepwise logistic regressions were performed with dichotomized PSP change (greater than or equal to one 10‐point category change). Receiver operator characteristic curve (ROC) analysis was also conducted (Hijian–Tilaki, 2013) (The ROC is a plot of the true‐positive rate against the false‐positive rate for the different possible cut‐offs of a diagnostic test). The accuracy of a test depends on how well it separates the group being tested into those with and those without the disease in question, or in this case, the improvement of social functioning as indicated by a <20‐point change in PSP score. Accuracy is measured by the area under the ROC curve (AUC); an AUC of 1 indicates a perfect test; 0.90–1 = excellent; 0.80–0.90 = good; 0.70–0.80 = fair; 0.60–0.70 = poor; and 0.500.60 = fail. As an alternative test of accuracy, an absolute PSP score of >70 (“not more than mild functional impairment”) at endpoint was used as the dependent variable.

## 3 RESULTS

### 3.1 Correlation between PSP change and change in other scales

The highest correlations with change in PSP total score were found for change in PANSS total score (Spearman's *r* = 0.607), followed by change in PANSS general psychopathology subscore (*r* = 0.579), and change in CGI‐S (*r* = 0.569). Correlations between change in PSP and the remaining PANSS subscales and Marder factor scores were mostly high or moderate. Weaker correlations were found between change in PSP score and change in quality of sleep, daytime drowsiness, the SF‐36 mental and physical components, ESRS total score, and treatment satisfaction at endpoint (Figure S1).

### 3.2 Power of PSP to predict CGI‐S change

**FIGURE 1:**
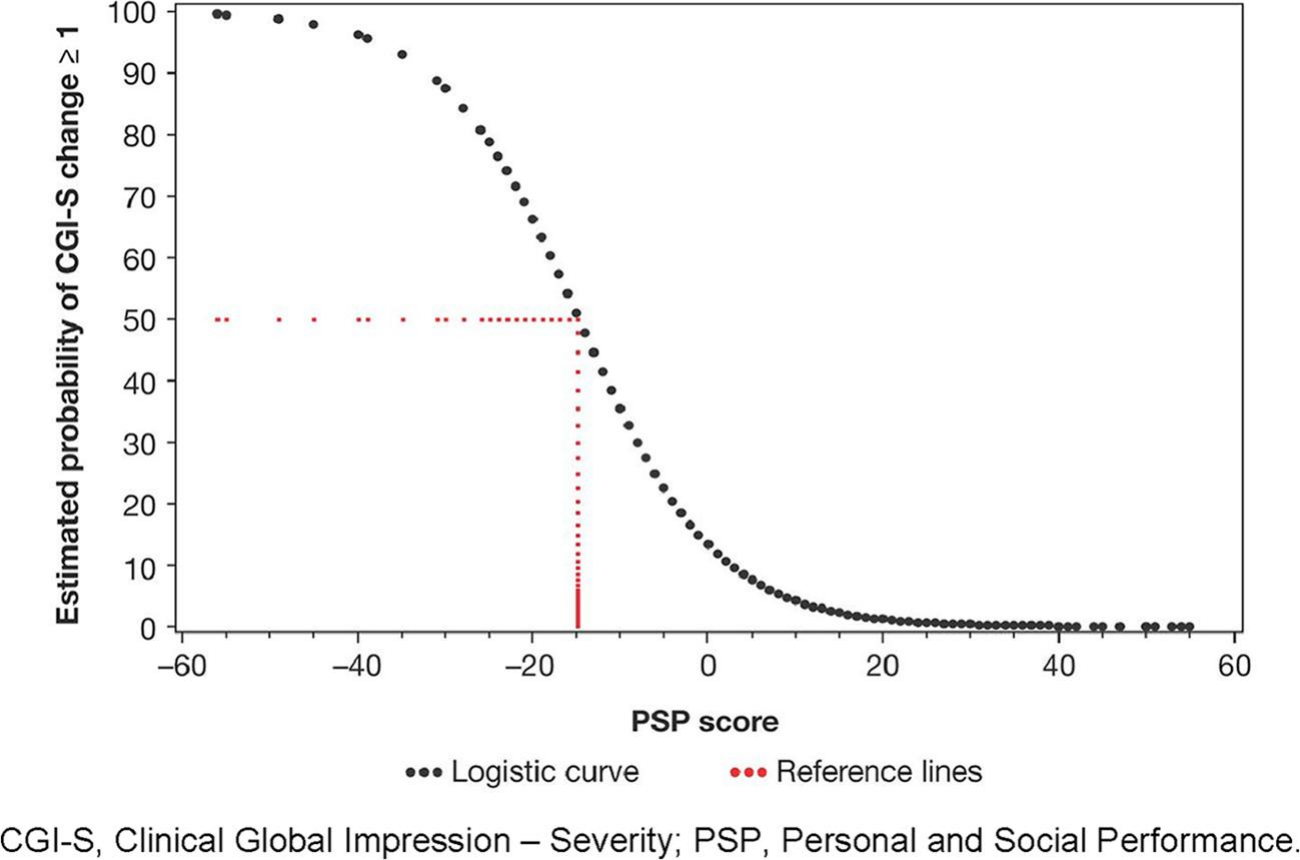
Relationship between PSP change and probability of a CGI‐S ≥1. CGI‐S, Clinical Global Impression–Severity; PSP, Personal and Social Performance

Logistic regression analysis showed change in PSP to be a highly significant predictor for change in CGI‐S (<1 vs. ≥1) (Figure 1). ROC analysis revealed that when no change in PSP score was observed, there was only a 10% (i.e., low) probability that the patient would show deterioration in disease severity as measured by a change in CGI‐S of ≥1. When there was a change of −32 in PSP score, there was a 90% (i.e., high) probability that the patient would show deterioration in disease severity as measured by a change in CGI‐S score of ≥1. The optimal cut‐off was estimated at a PSP change of –15 points; a smaller decrease or an increase in PSP suggests stability or improvement on the CGI‐S scale. The AUC for this analysis was 0.850, indicating a good test.

**FIGURE 2:**
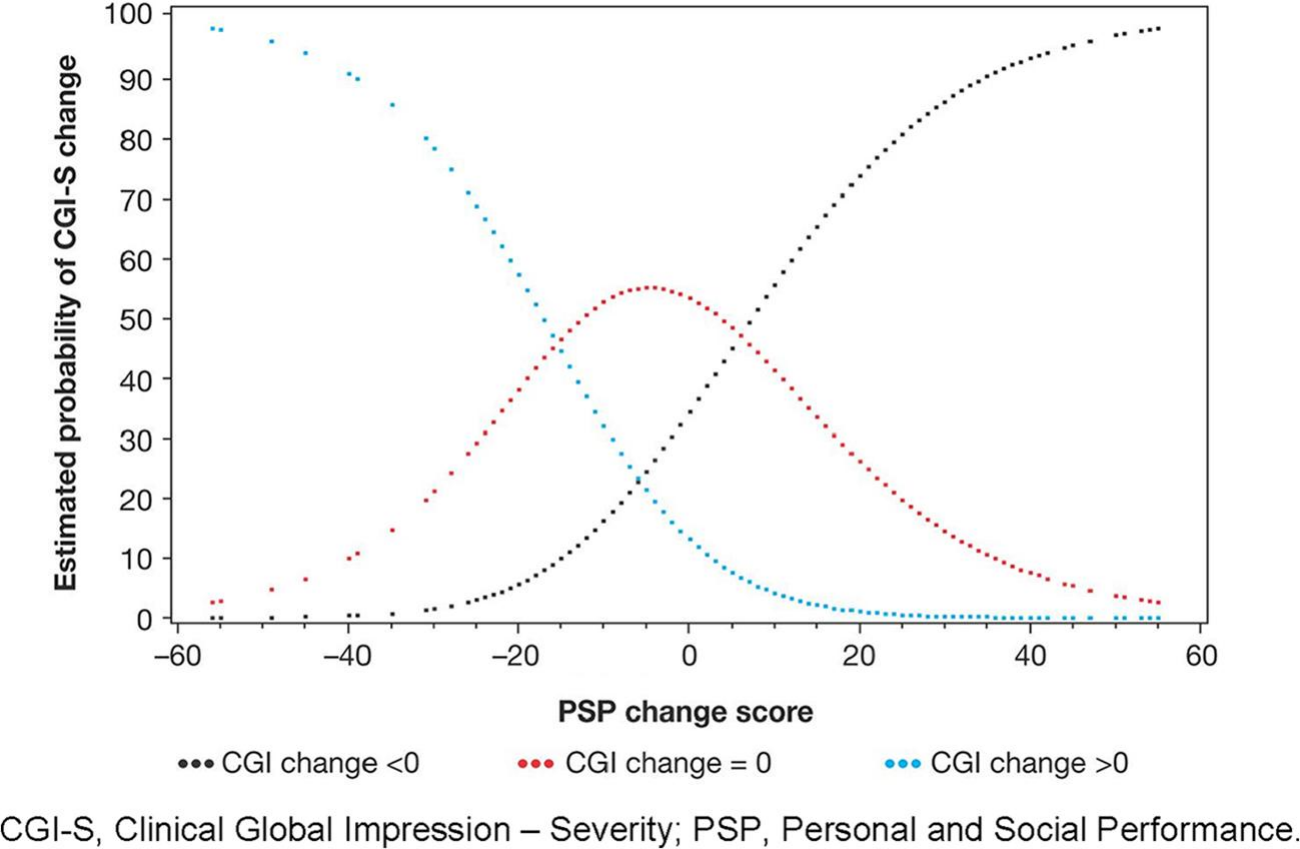
PSP change as a predictor of CGI‐S change (<0, 0, >0) CGI‐S, Clinical Global Impression–Severity; PSP, Personal and Social Performance

Discriminant analysis (Figure 2) enabled the estimation of two cut‐off values for change in PSP score to predict change in CGI‐S trichotomy (<0, 0, and >0). A decrease of ≥16 points on the PSP scale indicated the highest probability that disease severity as measured by the CGI‐S would also deteriorate; an increase of ≥6 points on the PSP scale indicated the highest probability that disease severity as measured by the CGI‐S scale would also improve. Consequently, PSP changes from −15 to 5 corresponded with no change in CGI‐S in most cases. The percentages of patients who were correctly predicted to belong to each of the three CGI‐S classes (<0, 0, and >0) were 70%, 66%, and 25%, respectively, with a total percentage correctly predicted of 63%.

### 3.3 Power of PSP to predict change in PANSS scores

PANSS total scores were categorized into three classes: (1) large improvement (a decrease of 20 points in PANSS total score), (2) moderate improvement (a decrease of 1–20 points in PANSS total score), and (3) no improvement (no change in PANSS total score), based on the 20‐points change that was deemed significant in nonacute patients. Discriminant analysis enabled cut‐off values for the PSP change scores associated with these PANSS categories to be estimated at 6 and 27; a change of ≥27 PSP points corresponded with a “large improvement” in PANSS total score, while a PSP change of ≤6 points corresponded with “no improvement” in PANSS total score. The predictive power of change in PSP to predict change in PANSS total score was less than the power of change in PSP to predict change in CGI‐S.

### 3.4 PSP change explained in a multivariate manner

**FIGURE 3:**
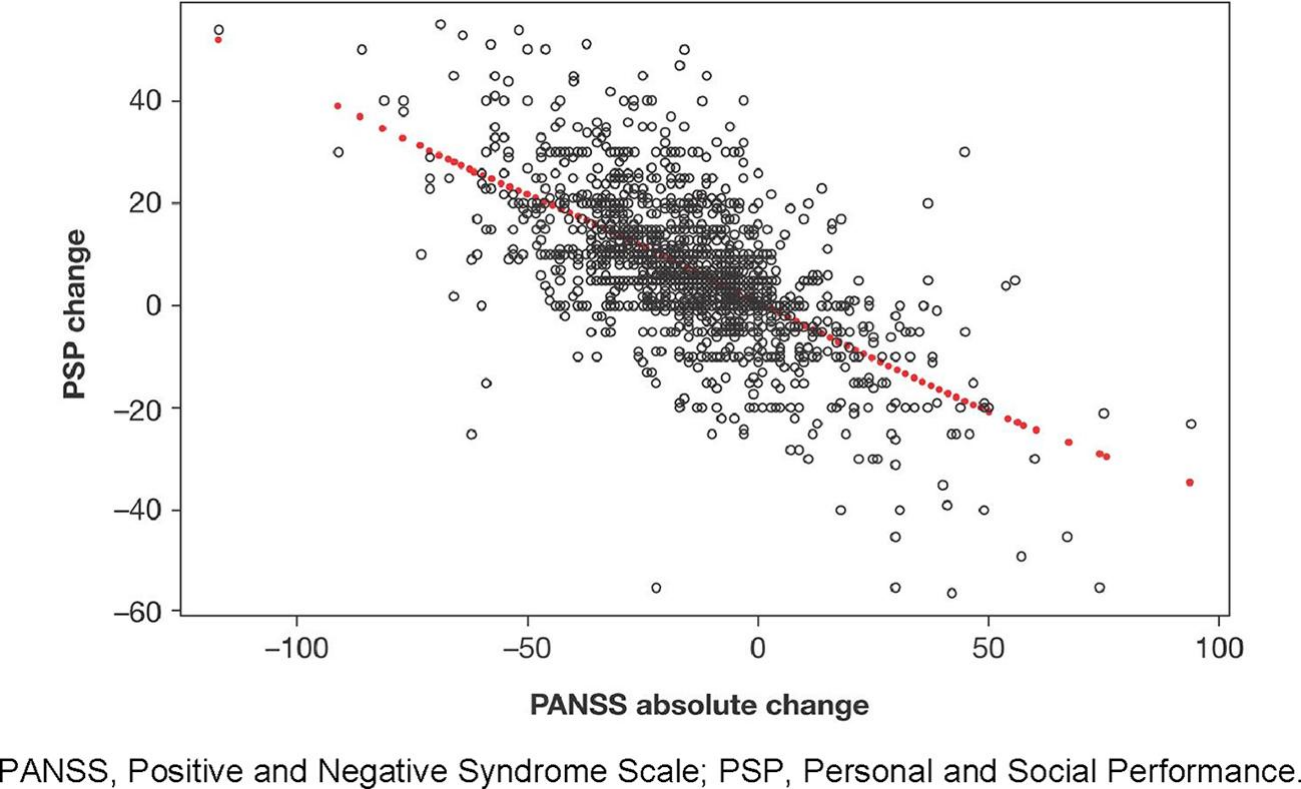
Relationship between PSP absolute change and PANSS absolute change. PANSS, Positive and Negative Syndrome Scale; PSP, Personal and Social Performance

Separate stepwise linear regression analyses were conducted for CGI‐S change alone, PANSS total score change alone, and both CGI‐S change and PANSS total score change, with change in PSP score as the dependent variable. *R* ^2^ for these analyses were 0.35, 0.39, and 0.44, respectively, where *R* ^2^ represents the goodness‐of‐fit of the trend line (how close the data are to the fitted regression line) and ranges from 0 (the model explains none of the variability in the data) to 1 (the model explains the total variability in the data). When all scales were combined and background characteristics were added in a multivariate analysis, significant relationships in the expected direction were found for PSP score change and change in PANSS total score (Figure 3), change in CGI‐S, change in SF‐36 Mental Component score, treatment satisfaction at endpoint, PSP score at baseline, and the number of previous psychiatric hospitalizations (Table 2). All of these additional factors raised the explained variation in PSP change to an *R* ^2^ of only 0.55, meaning that a large proportion could still not be explained. No significant additional contributions were found for changes in PANSS negative scale, PANSS positive scale, Marder Score for anxiety/depression, ESRS total score, sleep quality, daytime drowsiness, or the background variables of age, gender, and type of schizophrenia.

**TABLE 2:** Multivariate logistic regression analysis of the relationship between change in PSP score as the dependent variable and change in other scales and baseline characteristics as independent variables (*N* = 1,413, *R* ^2^ = 0.55)

Variable	Estimate	*p* value
Change in PANSS total score	−0.19546	<0.0001
Change in CGI‐S	−3.34430	<0.0001
Change in SF‐36 mental component score	0.09845	<0.0001
Treatment satisfaction at endpoint	−1.94259	<0.0001
PSP score at baseline	−0.30383	<0.0001
Number of previous psychiatric hospitalizations	−0.14972	0.0005

Abbreviations: CGI‐S, Clinical Global Impression–Severity; PANSS, Positive and Negative Syndrome Scale; PSP, Personal and Social Performance; SF‐36, Short‐Form 36.

As sensitivity analyses, the change in PANSS total score and change in CGI‐S were replaced by the three PANSS subscale scores (PANSS positive, PANSS negative, and PANSS general psychopathology) in separate regression models, plus the addition of changes in SF‐36 Mental Component score, quality of sleep, ESRS total score, and treatment satisfaction at endpoint. Although most of these factors were highly statistically significant, the *R* ^2^ in these models was ≤0.41, and thus provided no improvement compared with the model in Table 2.

An alternative logistic regression was carried out in a similar stepwise manner, to explain a ≥10‐point improvement in PSP total score. The univariate relationships between the dichotomous PSP change (i.e., improvement ≥10 points) and all measurement scales are shown in Table 3. As expected, all relationships were highly significant and in the expected direction. The best explaining power by means of the proportion correctly predicted or the AUC was found for the change in PANSS total and subscale scores and change in CGI‐S. SF‐36 Physical and Mental Components scores, sleep quality or daytime drowsiness, and ESRS total score were also significant predictors of improvement in PSP (Table 3). When all scales were combined in a stepwise manner and background characteristics were added to a multivariable model, findings followed a similar pattern as seen with the linear regression analyses (Table 4).

**TABLE 3:** Univariate logistic regression analysis of the relationship between a ≥10‐point improvement in PSP total score as the dependent variable and change in other scales and baseline characteristics as separate independent variables

Scale (change)	Estimate	*p* value	OR (95% CI)	n	AUC	Correctly predicted (%)
PANSS total score	−0.0755	<0.0001	0.927 (0.919–0.935)	1.646	0.805	73.7
PANSS positive subscore	−0.1826	<0.0001	0.833 (0.813–0.854)	1.646	0.738	69.9
PANSS negative subscore	−0.1812	<0.0001	0.834 (0.815–0.854)	1.646	0.755	70.0
PANSS general subscore	−0.1340	<0.0001	0.875 (0.861–0.888)	1.646	0.792	73.3
CGI‐S	−1.1166	<0.0001	0.312 (0.272–0.357)	1.647	0.770	70.2
SF‐36 mental component score	0.0629	<0.0001	1.065 (1.054–1.076)	1.533	0.697	66.6
SF‐36 physical component score	0.0535	<0.0001	1.055 (1.039–1.071)	1.533	0.599	61.8
Sleep quality	0.1585	<0.0001	1.172 (1.127–1.219)	1.544	0.624	63.2
Daytime drowsiness	−0.1253	<0.0001	0.882 (0.852–0.914)	1.547	0.619	60.7
ESRS total score	−0.0723	<0.0001	0.930 (0.908–0.953)	1.643	0.579	62.0

Abbreviations: AUC, area under the curve; CGI‐S, Clinical Global Impression–Severity; CI, confidence interval; ESRS, Extrapyramidal Symptoms Rating Scale; OR, odds ratio; PANSS, Positive and Negative Syndrome Scale; PSP, Personal and Social Performance; SF‐36, Short‐Form 36.

**TABLE 4:** Multivariate logistic regression analyses of the relationship between ≥10‐point improvement in PSP total score as the dependent variable and change in other scales and baseline characteristics as independent variables

Variable	Estimate	*p* value	OR (95% CI)
Change in PANSS total score	−0.0383	<0.0001	0.962 (0.952–0.973)
Change in CGI‐S	−0.5968	<0.0001	0.551 (0.457–0.664)
Change in SF‐36 mental component score	0.0314	<0.0001	1.032 (1.019–1.045)
Satisfaction at endpoint	−0.5230	<0.0001	0.593 (0.494–0.711)
Total PSP score at baseline	−0.0524	<0.0001	0.949 (0.939–0.959)
Number of previous psychiatric hospitalizations	−0.0625	0.0002	0.939 (0.909–0.971)

Abbreviations: CGI‐S, Clinical Global Impression–Severity; CI, confidence interval; OR, odds ratio; PANSS, Positive and Negative Syndrome Scale; PSP, Personal and Social Performance; SF‐36, Short‐Form 36.

### 3.5 Relationship between endpoint PSP, baseline variables, and other measures

The endpoint PSP score was dichotomized at 70 points because patients with PSP scores >70 are characterized as having only mild functional deficits (Kozma et al., 2010). A total of 16% of patients had a PSP score >70 at baseline, while 35% achieved the cut‐off score of >70 at endpoint (Kozma et al., 2010). There was a significant inverse relationship between achieving an endpoint PSP score >70 and changes in PANSS negative score, CGI‐S, and treatment satisfaction at endpoint. There were also significant inverse relationships between achieving an endpoint PSP score >70 and: gender, a diagnosis of disorganized or catatonic/residual‐type schizophrenia, and number of previous psychiatric hospitalizations. There was a significant positive relationship between change in the SF‐36 Mental Component score and change in PSP score (Table 5).

**TABLE 5:** Logistic regression analysis of the relationship between PSP Score >70 at endpoint and other variables

Variable	Estimate	*p* value	OR (95% CI)
Change in PANSS negative subscore	−0.0308	0.0252	0.970 (0.944–0.996)
Change in CGI‐S	−0.4789	<0.0001	0.619 (0.531–0.722)
Change in SF‐36 mental component score	0.00868	0.1185	1.009 (0.998–1.020)
Treatment satisfaction at endpoint	−0.6755	<0.0001	0.509 (0.432–0.600)
Gender	−0.2554	0.0432	0.775 (0.605–0.992)
Schizophrenia subtype diagnosis			
Disorganized	−0.5904	0.0531	0.554 (0.305–1.008)
Undifferentiated	−0.2783	0.1740	0.757 (0.507–1.131)
Catatonic/residual	−0.9408	0.0013	0.390 (0.220–0.693)
Number of previous psychiatric hospitalizations	−0.0494	0.0007	0.952 (0.925–0.979)

CGI‐S, Clinical Global Impression – Severity; CI, confidence interval; OR, odds ratio; PANSS, Positive and Negative Syndrome Scale; PSP, Personal and Social Performance; SF‐36, Short‐Form 36.

## 4 DISCUSSION

The background for this study is the limited consensus on the definition and measurement of psychosocial functioning, and on impairment and deterioration of psychosocial functioning, despite it being a characteristic feature of schizophrenia that is crucial to the understanding and treatment of the disease (Burns & Patrick, 2007). In this context, we aimed to investigate whether the PSP scale could represent a consensus‐candidate tool for the measurement of psychosocial functioning in schizophrenia, and to investigate what clinical factors impact psychosocial functioning as measured by PSP.

The present analysis showed that broad measures of illness severity (e.g., CGI‐S, number of previous psychotic hospitalizations, and PANSS) were significantly associated with the PSP score. Two cut‐off values for PSP change were estimated to predict change in CGI‐S: a deterioration in PSP score of ≥16 points was associated with a deterioration in CGI‐S; an improvement of ≥6 points in PSP score was associated with improvement in CGI‐S. This left a PSP score of –15 to 5 associated with no change in CGI‐S. The PSP change cut‐off of ≥6 was close to the cut‐off of 7 reported by Nasrallah and colleagues to indicate a clinically meaningful improvement in PSP score (Nasrallah et al., 2008). With regard to the association between PSP and PANSS score, the PSP change cut‐off values of 6 and 27 (PSP improvement >27 points predicting a PANSS improvement of >20 points and PSP change <6 points predicting a PANSS deterioration ≥0 points) were in line with the data from the scatterplot for absolute PSP change and absolute PANSS change showing the expected inverse linear relationship between the two measures. These findings are also in line with those of Nasrallah and colleagues (Nasrallah et al., 2008).

It is important to understand how changes on a rating scale correspond with illness severity, which can be measured using instruments such as the CGI‐S. A linking study in patients with schizophrenia treated with cariprazine or risperidone showed that greater improvement on PANSS scales resulted in less severe disease state according to the CGI‐S (Leucht et al., 2019). In the current study, a discriminant validation of PSP as a more independent measure of a thus far underestimated treatment goal of everyday functioning in social and vocational reality after schizophrenia treatment was performed. The power of the PSP to predict PANSS total score change is somewhat smaller than the power for predicting CGI‐S change. These results suggest in that in both future studies and routine care it might be useful to assess the PANSS score alongside the PSP score, and the overlap with CGI‐S may make the PSP score a sufficient proxy for this measure when assessing psychosocial function.

Despite the demonstrated relationships between CGI‐S, PANSS score, and other clinical measures, linear regression analysis showed that only 45% of the change in PSP was explained by all the assessed scales and background factors. This suggests that PSP change is driven by factors related to patient role function other than those assessed by other outcome measures. Interestingly, none of the following made significant additional contributions to the variation in PSP change when including all scales and background factors in the model: PANSS negative; PANSS positive; Marder subscore for anxiety/depression; sleep quality or daytime drowsiness; age; gender; type of schizophrenia. From a clinical perspective, confirmation that a substantial component of variation in the PSP is not explained by other clinically relevant assessments suggests that doctors should be utilizing this measure in order to fully assess changes in psychosocial function.

As relationships may be masked by the strong influence of the broad CGI‐S and PANSS total score measures, further linear regression was applied, excluding these measures. Their exclusion from the linear regression models and replacement with the individual PANSS subscores did not result in a greater predictive power from these models, confirming that a significant proportion of the PSP score was not explained by the included factors.

When a different approach was used (univariate logistic regression analysis with PSP improvement ≥10 points as the independent variable and various measurement scales as separate dependent variables), further variables were revealed that had weaker but statistically significant relationships to PSP change. Associations were found between: PSP improvement ≥10 points and improvements in PANSS scores (total, positive, negative and general psychopathology components); change in CGI‐S; change in daytime drowsiness; change in ESRS total score; change in SF‐36 mental and physical component scores; and change in sleep quality. Multivariate stepwise logistic regression showed that the relationship of change in PSP score with the following remained significant: PANSS total score, CGI‐S, or SF‐36 Mental Component score; treatment satisfaction at endpoint; baseline PSP score; and number of previous psychiatric hospitalizations.

Achieving a PSP score ≥71 is a clinically important goal, and has been reported to double the probability of a schizophrenia patient gaining employment, reflecting achievement of a major life goal (Dirani, Kozma, Mao, Amatniek, & Canuso, 2008). In general, a PSP score of >70 is achieved by patients having only mild functional impairment in all (or at least most) of the PSP categories, suggesting that overall functional impairment in the four domains measured needs to be mild to nonexistent to achieve this PSP score (Kozma et al., 2010). Multivariate logistic regression analysis of the dichotomized outcome of a PSP score >70 at endpoint showed that improvements in PANSS negative, CGI‐S, and treatment satisfaction at endpoint were associated with achieving only minor psychosocial impairment. Male gender, diagnosis of disorganized or catatonic/residual schizophrenia, and the number of previous psychiatric hospitalizations were associated with reduced likelihood of achieving only minor impairment. The effect of negative symptoms may be particularly important, with recent data suggesting that negative symptoms contribute to impaired functioning (Fervaha, Foussias, Agid, & Remington, 2014; Fulford et al., 2013; Mancuso, Horan, Kern, & Green, 2011; Ventura et al., 2015; Ventura, Hellemann, Thames, Koellner, & Nuechterlein, 2009). An improvement in PANSS negative subscale score was associated with achieving functional recovery (a PSP score >70), while an improvement in PANSS total score was more strongly associated with achieving a 10‐point increase in PSP score.

Improved psychotic symptoms (PANSS) and disease severity (CGI‐S) were associated with improvements in function as measured by the PSP scale; however, functional improvement was also related to improvements in a large number of other characteristics including improved patient treatment satisfaction, improved ESRS total score, improved overall mental and physical health (SF‐36 Mental and Physical Components), and improved sleep quality. All associations between function (PSP) and other measures were in the expected direction (i.e., improvements in patient functioning lead to improvements in other psychosocial and clinical domains and vice versa). The relationship with ESRS score should be noted, because the relationship between extrapyramidal side effects and functioning is often overlooked in the clinical setting.

Despite significant associations with this diverse range of factors, linear regression revealed that only 55% of the change in PSP could be explained by these measures alongside various baseline patient characteristics. It is acknowledged that some of the factors influencing social functioning in real life were not considered in the present study. These might include clinical (e.g., cognition) and nonclinical factors (e.g., personal factors such as resilience, and contextual factors, such as stigma, the incentives of financial and/or practical family support, and engagement with healthcare services) (Galderisi et al., 2014; Strassnig et al., 2015). A more recent study does show that functional outcomes, as measured by PSP, are related to both sociodemographic variables such as age and age at onset of schizophrenia and to nonclinical variables such as level of education, resilience, coping styles, and service engagement (Rossi et al., 2017). It is important to realize that this 55% is also likely to contain a number of (unidentified) random factors, so the actual amount of PSP change explained by other scales is likely to be smaller still. The validity of the PSP scale as a measure is indicated not only by its association with numerous other important scales, but also by the fact that a large proportion of PSP change cannot be explained by these scales, suggesting that unique elements of functional recovery are being assessed by the PSP scale.

Ideally, the discriminant analysis using the change in PANSS total score to predict change in the clinical impression scores (large, moderate, or no improvement on the CGI‐S) would have been carried out using percentage changes instead of absolute changes to support interpretation of the data. Unfortunately, the percentage changes were difficult to interpret as they were highly skewed. In a recent study, CGI‐S outcomes were shown to change in a corresponding manner regardless of whether relative or absolute changes in PANSS‐Factor Score for Negative Symptoms (FSNS) scores were used (Leucht et al., 2019) It is therefore unlikely that using percentage changes would have significantly changed the results of the current analysis. Other limitations of this study include the relatively short study duration and the lack of a comparison group in this single‐arm trial. Only PSP scores at baseline and 6 months after the start of paliperidone ER treatment were included in the analyses, while shorter intervals might be perceived as more relevant by patients and clinicians (Nicholl et al., 2010). In addition, several known predictors of cognitive functioning, such as neurocognition, were not measured in depth in the current study (Bell, Tsang, Greig, & Bryson, 2009; Bowie, Depp, & McGrath, 2010). A more detailed analysis may have resulted in a better understanding of the applicability of our results.

In conclusion, the results of this analysis demonstrate that not only severity of illness and symptoms (as measured by the CGI‐S scale and PANSS, respectively), but also other factors (as measured using the PSP scale) contribute to patient functioning. Addressing psychopathology (as measured by PANSS total score) may help to improve patient functioning in general, but specific attention should be paid to negative symptoms when aiming to minimize functional impairment. A specific finding of this analysis was that factors beyond clinical symptoms (i.e., mental and physical health, extrapyramidal side effects, and sleep quality) reflect relevant contributors to patient functioning when analyzed through separate logistic regression and should, therefore, be evaluated and addressed on a regular basis. Additionally, this analysis provides useful information on the amount of change needed in psychotic symptoms or disease severity to achieve a clinically meaningful improvement in patient functioning, and, thereby, may inform relevant treatment decisions. The study also provides additional insights into the correlation between frequently used clinical scales (such as the PANSS and CGI‐S scale) and patient functioning. The PANSS Marder five factors scale, also known as the PANSS‐FSNS, is widely used, enabling comparison with former studies in paliperidone ER treatment and other antipsychotic drugs (Leucht et al., 2019). For future studies, the PANSS consensus five factors, which has been used to evaluate clinical remission in schizophrenia (Pinna, Bosia, Cavallaro, Carpiniello, & Cagliari Recovery Group Study, 2014; Wallwork, Fortgang, Hashimoto, Weinberger, & Dickinson, 2012) or the more recently developed four‐factor PANSS consensus scale (Chen et al., 2020) could also provide clinically relevant insights.

The PSP scale may, therefore, be a valuable additional tool for assessing patient functioning as it encompasses a number of psychosocial and clinical factors; knowledge of specific contributors to changes in patient functioning may also help to better target symptoms to optimize outcomes.

## CONFLICT OF INTEREST

Roland Vauth has received funding for research, advisory board and sponsored lectures in the last 3 years from: Janssen‐Cilag, Otsuka, Lundbeck, Eli‐Lilly, and the Swiss National Fund. Bernardo Carpiniello has received grants as a consultant and/or as a speaker at congresses/scientific meetings from: Janssen‐Cilag Italy, Lundbeck Italy, and Otsuka Italy and has received research funding from Lundbeck Italy. Jacek Turczynski has participated in clinical trials sponsored by Servier, Sanofi, Janssen‐Cilag, Eli Lilly, AstraZeneca, Cephalon, Otsuka, Roche, Sunovion, Eisai, Lupin, and Lundbeck and received grants and speaker fees from Pfizer, Bristol‐Myers Squibb, Eli Lilly, LEKAM pharmaceuticals, Polpharma, and Krka pharmaceuticals. Mikhail Ivanov has no conflict of interest. Pierre Cherubin is an employee of Janssen‐Cilag France and a member of EMEA Medical Affairs at Jansen‐Cilag France. Marjolein Lahaye is an employee and a member of Medical Affairs EMEA at Janssen‐Cilag BV. Andreas Schreiner is a former employee of Janssen‐Cilag GmbH and is a shareholder in Johnson & Johnson. He was a member of Medical Affairs EMEA at Janssen‐Cilag GmbH at the time of the project conduct and publication development.

## Supporting information

Supplementary Material 1

Supplementary Material 2
