# Supplementary material for: Relationship between clinical outcomes measures and personal and social performance functioning in a prospective, interventional study in schizophrenia
Source: Int J Methods Psychiatr Res. 2020 Dec 23;30(2):e1855. doi: 10.1002/mpr.1855 (PMC8170566; doi:10.1002/mpr.1855)
Supplement: Supplementary file 1 — Supplementary Material 1 [file MPR-30-e1855-s002.docx]

**Supplementary Table 1.** Summary of Outcome Measure Scales Used

| **Scale** | **Description** | **Categories** | **Scoring** | **Reference** |
| --- | --- | --- | --- | --- |
| CGI | Provides an overall clinician-determined measure that takes into account all available information, including knowledge of the patient’s history, psychosocial circumstances, symptoms, behaviour, and the impact of the symptoms on the patient’s ability to function. | Two companion one-item measures evaluating the severity of psychopathology from 1 to 7 (CGI Severity) and the change from the initiation of treatment on a similar seven-point scale (CGI Improvement) | 7-point scale.  CGI Severity: How mentally ill is the patient? 1=normal, not at all ill; 2=borderline mentally ill; 3=mildly ill; 4=moderately ill; 5=markedly ill; 6=severely ill; 7=among the most extremely ill patients.  CGI Improvement: compared with baseline this patient’s condition is: 1=very much improved since the initiation of treatment; 2=much improved; 3=minimally improved; 4=no change from baseline; 5=minimally worse; 6= much worse; 7=very much worse. | Busner and Targum, 2007 |
| PANSS | The PANSS is a drug-sensitive instrument that scores positive and negative symptoms and assesses their relationship to one another and to global psychopathology. | PANSS includes 30 items assessing positive symptoms (7), negative symptoms (7), and general psychopathology (16) | 7-point rating scale per symptom item:  1=absent; 2=minimal; 3=mild; 4=moderate; 5=moderate severe; 6=severe; 7=extreme.  The scores for the three scales are arrived at by summation of ratings across component items. The potential ranges are: 7 to 49 for the positive and negative scales, and 16 to 112 for the general psychopathology scale. | Kay et al., 1987  PANSS rating criteria |
| SF-36 | The SF-36 is designed to assess health and wellbeing in clinical practice and research, health policy evaluations, and general population surveys. | The SF-36 assesses 8 categories related to health and wellbeing: physical functioning, bodily pain, role limitations due to physical health problems, role limitations due to personal or emotional problems, emotional well-being, social functioning, energy/fatigue, and general health perceptions. | Categories are assessed by weighted scales which are transformed into a 0 to 100 scale. The lower the score the more disability. | Ware and Sherbourne, 1992  RAND.org |
| Treatment satisfaction | Subjects were interviewed at start and at the end of the main extension phase to assess their satisfaction with the current treatment |  | 5-point categorical scale, ranging from 1 [very good] to 5 [very poor] ( very good, good, reasonable, moderate or poor) |  |
| Sleep quality | A self-administered scale rates quality of sleep. |  | An 11-point categorical scale, scored over the previous 7 days:  0 [very badly] to 10 [very well] |  |
| Daytime drowsiness | A self-administered scale rates quality of daytime drowsiness. |  | A 11-point scale over the previous 7 days, measured from 0 [not at all] to 10 [all the time] |  |
| ESRS | ESRS assesses four types of DIMD: Parkinsonism, akathisia, dystonia, and tardive dyskinesia. | The total score is based on 17 items from the Parkinsonism examination (subscale II): tremor, gait and posture, postural stability, rigidity, expressive automatic movements, bradykinesia, akathisia. | The total score ranges from 0 to 102 across categories: tremor (0–48), gait and posture (0–6), postural stability (0–6), rigidity (0–24), expressive automatic movements (0–6), bradykinesia (0–6), akathisia (0–6). | Chouinard and Margolese, 2005. |

CGI, Clinical Global Impression; DIMD, drug-induced movement disorders; ESRS, Extrapyramidal Symptom Rating Scale; OR, odds ratio; PANSS, Positive and Negative Syndrome Scale; PSP, Personal and Social Performance; SF-36, Short-Form 36.

**References**

Busner J, Targum SG. The clinical global impressions scale: applying a research tool in clinical practice (2007) *Psychiatry* *(Edgmont)* 4:28–37.

Chouinard G, Margolese HC. Manual for the Extrapyramidal Symptom Rating Scale (ESRS) (2005) *Schizophr Res* 76:247–265.

Kay SR, Fiszbein A, Opler LA. The positive and negative syndrome scale (PANSS) for schizophrenia (1987) *Schizophr Bull* 13:261–276.

PANSS Rating criteria. Available from: http://egret.psychol.cam.ac.uk/medicine/scales/PANSS Accessed, April 2017.

RAND.org. Medical Outcomes Study: 36-Item Short Form Survey Scoring Instructions. Available from: <http://www.rand.org/health/surveys_tools/mos/mos_core_36item_scoring.html>. Accessed, January 2014.

Ware JE, Sherbourne CD. The MOS 36-item short-form health survey (SF-36). I. Conceptual framework and item selection (1992) *Med Care* 30:473–483.
