# Supplementary figures and images for: Relationship between clinical outcomes measures and personal and social performance functioning in a prospective, interventional study in schizophrenia
Source: Int J Methods Psychiatr Res. 2020 Dec 23;30(2):e1855. doi: 10.1002/mpr.1855 (PMC8170566; doi:10.1002/mpr.1855)

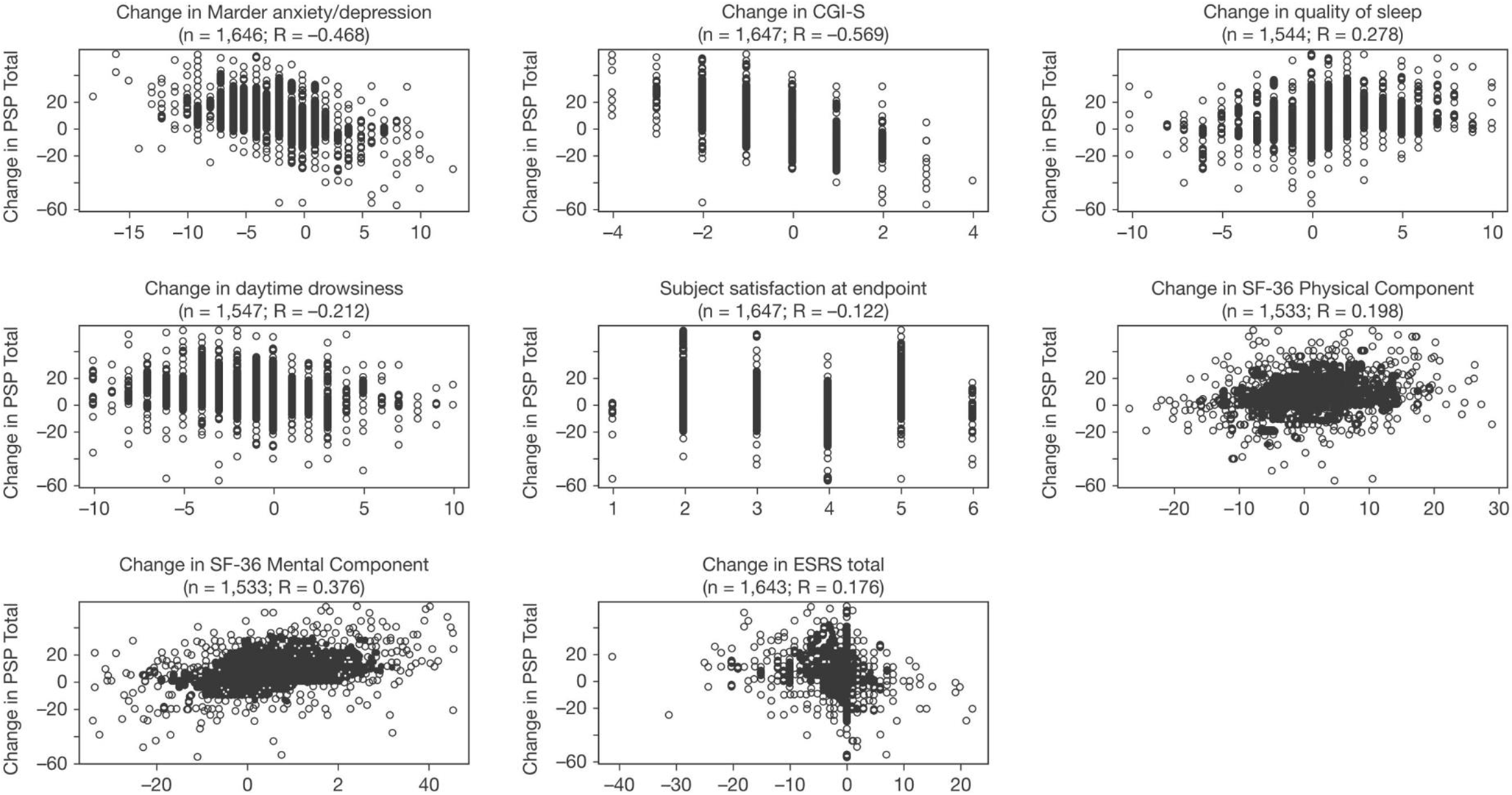

Supplement: Supplementary file 2 — Supplementary Material 2 [file MPR-30-e1855-s001.tif]
